# The Effectiveness of Intermittent Fasting, Time Restricted Feeding, Caloric Restriction, a Ketogenic Diet and the Mediterranean Diet as Part of the Treatment Plan to Improve Health and Chronic Musculoskeletal Pain: A Systematic Review

**DOI:** 10.3390/ijerph19116698

**Published:** 2022-05-30

**Authors:** Maria Cuevas-Cervera, Jose Javier Perez-Montilla, Ana Gonzalez-Muñoz, Maria Carmen Garcia-Rios, Santiago Navarro-Ledesma

**Affiliations:** 1 Department of Physiotherapy, Faculty of Health Sciences, Campus of Melilla, University of Granada, Querol Street, 5, 52004 Melilla, Spain; maaricuevass@correo.ugr.es (M.C.-C.); perezmontilla@correo.ugr.es (J.J.P.-M.); 2 Clínica Ana González, Avenida Hernan Nuñez de Toledo 6, 29018 Malaga, Spain; anagonzalez.fisioterapeuta@gmail.com; 3 Department of Physiotherapy, Faculty of Health Sciences, Campus of Granada, University of Granada, Av. de la Ilustración, 60, 18071 Granada, Spain; mcgrios@ugr.es

**Keywords:** musculoskeletal chronic pain, inflammation, fasting, caloric restriction, time restricted

## Abstract

Food strategies are currently used to improve inflammation and oxidative stress conditions in chronic pain which contributes to a better quality of life for patients. The main purpose of this systematic review is to analyze the effectiveness of different dietary strategies as part of the treatment plan for patients suffering from chronic pain and decreased health. PubMed, Web of Science, ProQuest, Scopus, Cumulative Index to Nursing & Allied Health Literature (CINAHL), Cambridge Core, and Oxford Academy databases were used to review and to appraise the literature. Randomized clinical trials (RCT), observational studies, and systematic reviews published within the last 6 years were included. The Physiotherapy Evidence Database (PEDro) scale, the PEDro Internal Validity (PVI), the Standard Quality Assessment Criteria for Evaluating Primary Research Papers from a variety of fields (QUALSYT), and the Quality Assessment Tool of Systematic Reviews scale were used to evaluate the risk of bias of the included studies. A total of 16 articles were included, of which 11 were RCTs and 5 were observational studies. Six of them showed an improvement in pain assessment, while two studies showed the opposite. Inflammation was shown to be decreased in four studies, while one did not show a decrease. The quality of life was shown to have improved in five studies. All of the selected studies obtained good methodological quality in their assessment scales. In the PVI, one RCT showed good internal validity, five RCTs showed moderate internal quality, while five of them were limited. Current research shows that consensus on the effects of an IF diet on pain improvement, in either the short or the long term, is lacking. A caloric restriction diet may be a good long term treatment option for people suffering from pain. Time restricted food and ketogenic diets may improve the quality of life in chronic conditions. However, more studies analyzing the effects of different nutritional strategies, not only in isolation but in combination with other therapies in the short and the long term, are needed.

## 1. Introduction

Chronic pain has an enormous impact on a personal and socioeconomic level [1]. Its nature is not specific, meaning that the level of tissue damage is not related to the severity of the pain experience [2].

Chronic musculoskeletal pain is defined as pain that recurs for more than three months, has a significant impact on functionality, and causes emotional stress [3]. This type of pain usually appears after an illness or injury, however it is not understood as a symptom but as a characteristic of the condition [4]. Chronic pain can be classified as nociceptive or neuropathic [2], with the central and the peripheral nervous systems being affected by the multiple etiologies that cause pain [5,6]. It can vary greatly from one person to another and even within the same individual depending on the context [6,7]. In the US population, 37% suffer from chronic pain, which is much higher than any other disease. In the European Union, the prevalence is between 18.6% (Switzerland) and 45.6% (France) [2,3].

A prolonged neuroinflammation, which is a failure in the response of the immune system when helping to promote homeostasis, may result in chronic pain [8]. When this failure lasts for a period of time, it aggravates the damage and a chronic low grade inflammation (LGI) develops [9]. The presence of this chronic LGI suggests the existence of chronic musculoskeletal pain as well as peripheral sensitization, which ultimately leads to central sensitization [10]. LGI is considered to be a risk factor in chronic diseases [11]. Current research shows that the food regimen being followed has a direct impact on the immune system, which is activated by a high-fat diet, leading to an increase in cytokine levels, and this has a direct impact on pain [12,13]. In addition, high glucose levels have been shown to facilitate central nervous system (CNS) sensitization [14]. A poor diet contributes to CNS sensitivity by producing necrotic cells, oxidative stress, and tissue damage in the body, thus producing Toll-like receptors. These receptors, when activated, trigger a series of inflammatory immune events and activation of the glial cell, which results in low-grade inflammation. Glial activity is capable of initiating an alteration in CNS sensitization through different mechanisms [14].

Recent research confirms that nutritional interventions mainly in the dietary pattern, the intake of specific nutrients, and the number of meals can provide significant relief for patients with chronic pain. This happens through a great diversity of molecular systems that support both neural function and plasticity, and they produce effects similar to antidepressants [15]. These improvements may be secondary to clinical benefits, and they may arise due to a dietary link with CNS sensitization and therefore hypersensitivity to pain [11,13,14]. Some of the most modern food strategies being researched to improve both health and quality of life, are intermittent fasting, caloric restriction, time restricted feeding, a ketogenic diet, and a Mediterranean diet [10,14,16]. Intermittent fasting (IF) promotes cell efficiency by reducing inflammation and oxidative stress. In turn, this decrease in the number of meals per day favors the increase of synaptic plasticity and it stimulates the creation of new neurons that develop from neuronal stem cells [17]. Intermittent fasting improves the effectiveness of interventions that are used to treat chronic pain, both functionally and mechanically, and it helps to decrease pain perception and to reduce central and peripheral inflammation [17].

One of the most widely used diets for weight loss is caloric restriction, which reduces the daily calorie intake without causing malnutrition. It is a nutritional strategy, which has been shown to play a crucial role in people’s longevity and health [18]. In the short and long term, it increases glucocorticoid levels, which consequently reduces depressive symptoms [15].

Time restricted feeding is the regulation of food intake over a period of time. It consists of maintaining a daily meal routine, with a certain number of hours designated to fasting and others to eating [19]. This strategy is a specific type of IF where 12–18 h of fasting duration is used depending on the source of the information [19,20]. The benefits obtained are similar to those achieved by the IF diet, and they include a balancing of the circadian rhythm, which means better synchronization and function of physiological processes [20].

The ketogenic diet (KD), which is a proven strategy for epilepsy, is characterized by maintaining the carbohydrate intake to below 50 g per day or no more than 10% of the total energy ingested [21,22]. Improvements in pain and inflammation have been reported after using a KD. This can be explained through ketolytic metabolism, which produces an improvement in the mitochondrial coenzyme Q couple and in the cytoplasmic glutathione couple as well as a decrease in reactive oxygen species [23].

The Mediterranean diet (MD)is characterized by the frequent consumption of olive oil, fresh or dried fruit and vegetables, a moderate amount of fish, unrefined cereals, dairy and meat, and many condiments and spices, which are all accompanied by wine or its infusions; and, therefore, it is rich in nutrients and bioactive compounds with anti-inflammatory effects [10]. Many different conditions, such as cardiovascular disease [11,12], type 2 diabetes mellitus [13,14], dementia [15], and various types of cancer [16,17,18] can benefit from an MD.

Furthermore, other factors have to be taken into account such as the microbiota–brain axis, which is the relationship between the microbiota and the CNS. The existence of intestinal inflammation produces an inflammatory response which may reach the CNS and lead to chronic pain and its intensification [10]. On the other hand, changes in behavior and lifestyle may cause changes in the brain, which are associated with chronic pain. These changes may possibly be modifiable and reversible [17].

An essential part of attaining musculoskeletal well-being is the nutritional and dietary behavior of the patient in order to avoid the alteration or perpetuation of chronic musculoskeletal pain conditions. Therefore, research on the nutritional strategies used and which strategies are considered to be useful for the improvement of health and quality of life in terms of chronic musculoskeletal pain and inflammation is of great importance.

Thus, the main goal of the study was to review current research focused on the efficacy of intermittent fasting, time restricted feeding, caloric restriction, and ketogenic and Mediterranean diets when applied to improve both health and chronic musculoskeletal pain. Furthermore, we assessed the methodological quality of the studies analyzed.

## 2. Method

### 2.1. Study Design

The present study is a systematic review of the literature on randomized clinical trials, observational studies, and systematic reviews. It was carried out following the criteria defined in the PRISMA declaration for the preparation and the development of systematic reviews and meta-analyses [24].

### 2.2. Search Strategy

Seven electronic databases were used for the bibliographic search from inception to March 2022 by two independent investigators (M.C.-C. and J.J.P.-M.): PubMed, Web of Science, ProQuest, Scopus, CINAHL, Cambridge Core, and Oxford Academy.

The research question followed the description of the components of the PICO strategy (Population: patients with chronic musculoskeletal pain for more than three months and older than 18 years of age; Intervention: reduction of the symptoms of chronic pain after the application of nutritional strategies; Comparison: application or not of food strategies; Outcomes: effectiveness of nutritional strategies in health and chronic pain).

A sensitive search strategy using relevant search terms that were developed from Medical Subject Headings (MeSH) and Descriptors in Health Sciences dictionaries (DeCS) was used, as shown in Table 1.

#### 2.2.1. Eligibility Criteria

The PECOS framework, as aforementioned, was followed to determine which studies were included in the present systematic review. Each study had to meet the following inclusion criteria:(i) published from January 2015 to March 2022, (ii) published in the English language and internationally peer-reviewed, (iii) classified as RCTs, observational studies, (iv) full text available, (v) adults over 18 years of age, and (vi) participants had to be suffering from chronic musculoskeletal pain for at least 3 months. The exclusion criteria were studies that (i) did not evaluate chronic pain, (ii) did not apply food strategies in people with chronic pain as treatment, and (iii) did not show results or interpretation of their data.

#### 2.2.2. Study Selection

All studies identified by the search strategy were screened using the eligibility criteria that were previously specified. The first stage of assessment involved the screening of titles and abstracts by two reviewers (M.C.-C. and J.J.P.-M.). The same reviewers undertook the second stage, screening the full text. In cases of disagreement, a decision was made by consensus or, when necessary, a third reviewer (S.N.-L.) was consulted.

#### 2.2.3. Data Extraction

Two independent reviewers (M.C.-C. and J.J.P.-M.) who were blinded to each other extracted the following relevant data from each study: study details (first author, year of publication), characteristics of participants, setting, pain condition, SE measuring instrument, outcome measures, duration of follow-up, and study design. If there was any discrepancy between reviewers, a third reviewer was consulted (S.N.-L.).

### 2.3. Deviation from the Protocol

The scope of the search included all aspects of research within the field of nutrition, such as macronutrients, micronutrients, foods, food groups, dietary supplements, diet analysis, diet patterns, applied diets, dietary behavior. However, this approach resulted in 24,500 eligible studies with evidence of a link between nutrition (dietary patterns, dietary supplements, specific nutrients, intravenous nutrient therapy, intramuscular vitamin injection, etc.) and chronic pain.

In order to increase the quality of the study, an extra search was carried out which included different etiologies of when non-musculoskeletal chronic pain appeared. Additionally, we also attempted to consider individuals’ overall dietary intake and eating habits. This provided a quantity of more than 13,000 articles. However, a review that includes so many articles with a broad nutritional focus would decrease readability and understandability. Therefore, it was decided to narrow down, and focus the review on chronic musculoskeletal pain. However, the scientific evidence on chronic musculoskeletal pain from other pathologies was reflected, which, even narrowing the search criteria, continued to appear, and we understood that it was important to add it given the similar clinical characteristics.

### 2.4. Assessment of Methodological Quality

#### 2.4.1. Assessment of the Methodological Quality of RCTs

The methodological quality of the RCTs was performed using the PEDro scale translated and adapted into Spanish. This scale allows the validity of the articles to be evaluated.

The PEDro scale examines, through 11 items, the external and the internal validity of a study. For each completed item, with the exception of the first, the scale adds 1 point to the total score; therefore, the global score of the PEDro scale has a range of 0 to 10 points [25]. The evaluation of the internal validity of the RCTs was carried out by means of the Internal Validity Score (PVI). This scale collects the items of the PEDro scale selected as the most representative for internal validity. The selected values are 2, 3, 5, 6, 7, 8, and 9, and they are classified as follows: Articles with a PVI between 9 and 10 are considered to have an excellent methodological quality; articles with a PVI between 6 and 7 are considered to have a high methodological quality; articles with a PVI between 4 and 5 are considered to have an average methodological quality; and articles with a PVI between 0 and 3 are considered to have a low methodological quality [25].

#### 2.4.2. Assessment of the Methodological Quality of Observational Studies

The methodological quality of the observational studies was performed using the Quality Assessment Tool for Observational Cohort and Cross-Sectional Studies scale.

This scale is made up of 14 items that allow us to examine and to determine the internal quality of an observational study. Each item, if it is fulfilled, adds a point to the global score, which ranges from 0 to14 points; where 0 means poor methodological quality and 14 good [26].

#### 2.4.3. Qualitative Synthesis of the Selected Observational Studies

The quality of the study depends on the score acquired. The evaluation of the methodology is determined as “high”, “average”, and “low” [26].

It is understood that naming a study as “good” quality means it tends to have a lower risk of bias and its results are treated as valid. When rated as “fair”, the possibility of bias is relative, and the results are insufficiently valid. Finally, a methodology classified as “bad” is understood as one that presents a significant risk of bias and poor validity [26].

## 3. Results

The results found in the different selected studies are detailed below.

### 3.1. Study Selection

The searches performed in the seven different databases yielded: PubMed studies, Web of Science studies, ProQuest studies, Scopus studies, CINAHL studies, Cambridge Core studies, and Oxford Academy studies.

All those that did not meet the aforementioned inclusion criteria were eliminated.

**Figure 1:**
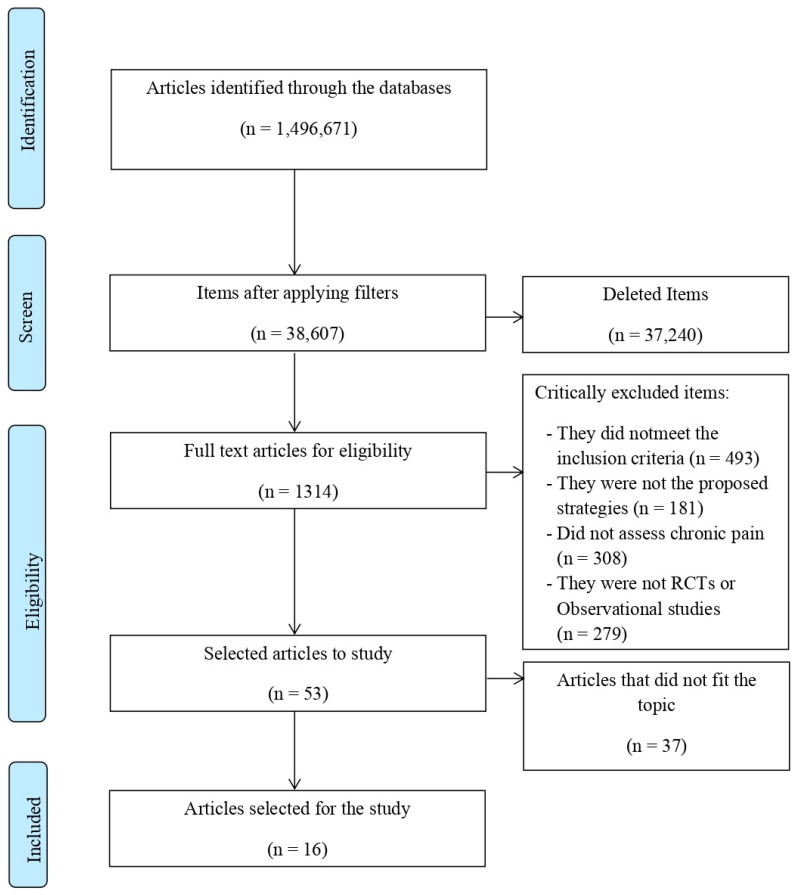
Flow diagram according to the PRISMA system. Study selection and exclusion process.

Finally, a total of 16 studies were selected, including 11 RCTs and 5 observational studies. The study selection process is shown in Figure 1 [24].

### 3.2. Assessment of Methodological Quality

A methodological quality assessment was carried out using the different scales with the results being shown in Table 2, Table 3 and Table 4. Development of the general characteristics of the selected studies is shown in Table 5.

### 3.3. Study Instruments

#### 3.3.1. Pain

The intensity, quality, and frequency of pain in the patients who participated in the application of dietary strategies were not reduced in the studies by Baurersfeld SP. et al. [29].

On the contrary, the studies carried out by, Holton KF. et al. [36] and Towery P. et al., [39] showed that the use of different types of food and nutrition significantly impacted the improvement of chronic pain symptoms in patients who suffered from it.

A decrease in pain levels was observed via SF-36 and AIMS2-2F [39]). In addition, there was a statistically significant association to adherence to the treatment plan, since those who did not adhere to the plan had no improvement in their pain symptoms (*p* = 0.025) [34,41,42].

#### 3.3.2. Inflammation

The concentration of slCAM-1, a soluble component of intercellular adhesion, which is a part of the inflammatory process, was studied and analyzed in different tissues and in structures in patients suffering from chronic pain and altered health. In the study by Stekovic S. et al. [33], they showed that there is no notable improvement in this variable.

On the other hand, the studies by Liu B. et al. [30] and Vadell AKE. et al. [34] stated that the application of dietary strategies can greatly decrease the levels of inflammation found in those suffering from chronic pain.

The levels of inflammation seen in tissues increased after the ingestion of food (*p* = 0.02) [30], and it was less during periods of fasting (*p* = 0.04) [32].The concentration of anti-inflammatory indicators (EPA and DHA) in the groups in which a treatment strategy had been applied increased (*p* ≤ 0.001 in both cases) [34].

#### 3.3.3. Quality of Life

The studies by Towery P. et al. [39] and Vadell AKE. et al. [34] showed that the application of dietary strategies for people suffering from chronic pain had a positive impact on quality of life.

A statistically significant association between diet and quality of life was found via the following questionnaires: a MED (*p* = 0.0001), WOMAC Scores (*p* = 0.01) [38], Health Questionnaires (*p* = 0.023), and General Questionnaires (*p* = 0.033) [39,40].

- Visual Analogue Scale (VAS): a scale used to measure pain. It consists of a horizontal or a vertical line that represents the pain intensity with a score from 0 to 10, where the number 0 is synonymous with “no pain” and 10 is synonymous with “very intense pain/worst pain imaginable” [43].
- SF-36: a tool for the evaluation of Health-Related Quality of Life (HRQoL). Scale applicable to any population. It contains 36 items that lead to the assessment of a positive and a negative quality of life. These questions are divided into eight scales in which physical function, physical role, bodily pain, general health, vitality, social function, emotional role, and mental health are questioned [44].
- Functional Assessment of Cancer Therapy (FACT-G): a quality of life assessment scale. This questionnaire is made up of 27 items that collect, on four scales, the values of physical state, family and social environment, emotional state, and functional capacity. Each item has a value of 0 to 4 points [45].
- Functional Assessment of Chronic Illness Therapy-Fatigue (FACIT-F): a questionnaire related to health and quality of life associated with chronic pain illnesses. It consists of 27 general questions that encompass physical well-being, family/social well-being, emotional well-being, and functional well-being [46].
- Functional Assessment of Chronic Illness Therapy-Trial Outcome Index (FACIT-TOI): a calculator for any illness, treatment or condition scale. An appraisal criterion that is normally used in clinical trials since it responds to physical and to functional changes [46].
- Fibromyalgia Survey Questionnaire (FSQ): a questionnaire for the evaluation of symptoms in people suffering from fibromyalgia. It incorporates the WPI scale and the modification of the SSS [47].
- Arthritis Impact Measurement Scales 2 (AIMS2-SF): a questionnaire that assesses physical, mental, and social conditions based on nine scales: mobility, physical activity, stroke, dexterity, household activities, pain, social activity, depression, and anxiety. Each scale is made up of four to seven items. The overall score ranges from 0 to 10 points [48].
- Numerical Pain Scale (NPRS): a one-dimensional tool for measuring pain in adults, consisting of 11 items. It is a version of the VAS scale that has scores from 0 to 10, with 0 being synonymous with “no pain” and 10 being “the most intense pain imaginable” [43].
- Western Ontario and McMaster Universities Osteoarthritis Index (WOMAC): a specific questionnaire for the measurement and the evaluation of pain, stiffness, and functional capacity in knee and hip osteoarthritic disease. It is composed of three scales (pain, stiffness, and functional capacity), which includes 24 items. The items are answered verbally using a scale which has five levels (0–4), ranging from little to a lot [49].
- EuroQoL-5D (EQ-5D):an assessment of the standard of living in Europe. It consists of five questions about mobility, personal care, day-to-day activities, pain/discomfort, and anxiety/depression [50].
- Health Assessment Questionnaire (HAQ): a questionnaire used to calculate the health of people with rheumatoid arthritis. It quantifies gastropathy, risk factors, and the risk of death in rheumatoid arthritis [51].
- Patient Global Assessment (PGA): one of the most used assessments in rheumatoid arthritis. It is based on a question related to general health or disease activity that has a numerical answer from 0 to 10 or 0 to 100 [52].
- Short-Form Health Outcome Survey (SF-12): a survey comprised of 12 questions that evaluates health in relation to quality of life [53].
- Knee Injury and Osteoarthritis Outcome Score (KOOS): this separately evaluates pain (9 items), symptoms (7 items), ADL function (17 items), sport and recreation functioning (5 items), and quality of life (4 items). Each item can be scored on a scale of 0 to 4 [54].
- Disease Activity Score on 28 joints (DAS28): a system to evaluate the activity of the disease and the treatment response in rheumatoid arthritis. The stipulated limit for an increase in treatment is 3.2 [51].
- Simple Disease Activity Index (SDAI): a system to evaluate, at any time or place, the activity of rheumatoid arthritis. Doctors can choose the evaluation tool that best fits the environment they find themselves in [55].
- Rheumatic Arthritis Impact of Disease (RAID): this evaluates pain, functional impairment, fatigue, sleep, and physical and emotional functioning in patients with rheumatoid arthritis. It is a validated tool that is highly trustworthy and sensitive to change [56].
- Healthy Eating Index 2015 (HEI-2015): a measuring tool to evaluate whether certain food groups follow the Dietary Guidelines for Americans (DGA). Each time a new DGA is published, the HEI is updated. In comparison to the 2010 update, the 2015 version included specific recommendations on the limitation of added sugars and saturated fats [57].

### 3.4. Intermittent Fasting

Intermittent fasting in healthy individuals [27,32] over a six month intervention period and in chemotherapy patients in the short term [29] can be well tolerated without causing adverse effects.

Fasting significantly reduced the levels of plasma insulin but left them unchanged if anti-oxidant supplementation was consumed [27].A decrease in the level of oxidative stress, no significant change in gene expression, but a trend in SIRT3 toward significance [27] was seen. On the other hand, an increase in insulin resistance was observed at the end of the fasting period [28].

In the short term (less than 12 weeks), intermittent fasting appears to improve quality of life and fatigue in healthy subjects [27] and in patients on chemotherapy [29].

A reduction in total weight was observed in subjects who had a decrease in lean muscle mass, although overall there was no significant change in the weight of subjects during intermittent fasting. Those who presented a decrease in lean muscle mass led to a reduction in total weight, however there was no significant change in the overall weight of subjects during intermittent fasting [28,32]. In the medium term (more than 12 weeks and less than 6 months), there was a significantly observable decrease in weight, fat [30,32], and serum non-esterified fatty acids [30]. After fasting, non-esterified fatty acids, M1-macrophages (CD40+) in adipose tissue, and M2-macrophages (CD163+) in muscle tissue had increased. Additionally, the changes in non-esterified fatty acids and in the mRNA of pan-macrophage marker CD68 in adipose tissue were positively correlated [30].

Inflammation markers, in serum (TNFα, IL6, and IL10) and in subcutaneous adipose tissue and skeletal muscle (CD68, CD40 and CD163), remained unchanged after the eating phases [30]. In addition, positive effects resulted in a reduction in sICAM-1 and DAS28-ESR [32,34]. The levels of low-density lipoprotein and metabolic regulator triiodothyronine were reduced after long-term intermittent fasting (more than 6 months) [32].

Moreover, changes in measurements, such as average dolorimetry, symptom score, tender point number, myalgic score, the Chalder Fatigue Scale, and significantly less Gulf War Illness symptoms, have been reported [36].

Intermittent fasting improved cardiovascular markers and b-hydroxybutyrate levels increased even on non-fasting days. On fasting days, pro-aging amino-acid methionine was periodically depleted, while polyunsaturated fatty acids were elevated [32].

On the other hand, an increase in LDL, plasma TNF-alfa, plasma cortisol, and systolic blood pressure, and a decrease in HDL, VO2 max, and the CANTAB score were produced by intermittent fasting, while avoiding physical activity and spending time resting in bed [28].

### 3.5. Time Restricted Feeding

The results obtained showed that levels of biomarkers HbA1 and fasting plasma glucose were reduced by 18% (*p* < 0.001) and 15% (*p* < 0.001), respectively, after the intervention of TRF. There was a significant improvement in the HOMA-β (*p* = 0.005) and HOMA-IR. (*p* = 0.02) scales along with a reduction in body weight and BMI [37].

In regards to the eating window, no significant differences were found when comparing results between groups (*p* = 0.62) [37].

After the intervention, an improvement in quality of life was produced in those that received TRF. The final rating on the SF-12 questionnaire was favorable (*p* < 0.001) [37].

### 3.6. Caloric Restriction

The level of adipocytes decreased in all groups (*p* = 0.01). A significant increase in the level of tissue inflammation after eating (*p* = 0.02) was found. These levels decreased with intermittent fasting (*p* ≤ 0.01) but not with caloric restriction. Body weight and body fat decreased (*p* < 0.05) [30].

Once the follow-up time period had ended, no significant changes were observed in glucose level (*p* = 0.956), HDL-C (*p* = 0.728), or LDL-C (*p* = 0.726) [30].

### 3.7. Ketogenic Diet

A reduction in glucose levels (*p* = 0.001) and an increase in oxidative metabolic stress, at the cellular level, were observed in patients who received radiation or chemotherapy after having completed 90 days of the intervention [33].The levels of cholesterol (*p* = 0.43), triglycerides(*p* = 0.42), HDL-C (*p* = 0.39), LDL-C (*p* = 0.45), TC:HDL-C ratio (*p* = 0.28), TG:HDL-C ratio (*p* = 0.94), thyroid hormone, and electrolytes showed no difference between groups. After the intervention, lactate values decreased significantly after 12 weeks of intervention(*p* = 0.02) [31,35].

A decrease in sugar consumption (*p* < 0.0001), daily calories (*p* < 0.01), weight loss (*p* < 0.001), BMI (*p* < 0.001), body fat (*p* = 0.03), and the sensation of appetite/hunger were all observed after 12 weeks of a KD intervention (*p* = 0.02). In addition, blood lipids were improved [32,34,36].

### 3.8. Mediterranean Diet

Subjects with a higher adherence to a Mediterranean diet, of unspecified duration, scored better results in quality of life scales [39,41,42], however a smaller group reported a non-significant variation in those scores [42]. Levels of pain, functional disability, wellness of sleep, physical and general well-being, and pain location and severity were significantly lower in subjects who followed a Mediterranean diet [39,41,42], but pain frequency and articular stiffness were exceptions [38,41]. There was a reduction in the mass of those subjects who had strictly adhered to a Mediterranean diet but this was not a significant conclusion [42]. A Mediterranean diet is considered to be a protective factor against arterial hypertension [40] and depression [38,41].

The strict adherence to a Mediterranean diet is significantly related to a healthier score in quality of life and chronic disease symptom scales and questionnaires [38,40,41,42].

## 4. Discussion

The objective of this study was to systematically review the existing scientific evidence on the efficacy of dietary strategies applied as part of the treatment of chronic musculoskeletal pain and health. As a result, 16 articles were selected of which 11 were RCTs and 5 were observational studies.

Within the selected studies, six of them showed an improvement in chronic pain symptoms in response to a specific food strategy. These strategies included: a low glutamate diet for Gulf War Syndrome [36], the diet recommended in the HEL-2015 Guide for different age groups [58], a vegetarian/vegan diet, intermittent fasting, caloric restriction, food restricted time, a Mediterranean diet, a plant-based diet, a diet rich in polyunsaturated fatty acids, antioxidant vitamins and minerals, fruits and fibers, a FODMAP diet, and the elimination of monosodium glutamate for patients suffering from fibromyalgia, rheumatoid arthritis, chronic musculoskeletal pain, and neuropathic pain [10,59,60,61]. There were two studies where no decrease in the perception of pain intensity and/or frequency were found [29,39]. With regard to the levels of inflammation, four studies found it decreased through the combination of intermittent fasting and caloric restriction for people diagnosed with obesity [30], through anti-inflammatory nutrition for rheumatoid arthritis sufferers [34], and through intermittent fasting, caloric restriction, a vegan/vegetarian diet and a Mediterranean diet for people with chronic musculoskeletal pain, fibromyalgia and/or rheumatoid arthritis [60,61]. Only one study showed no improvement in the levels of inflammation [32].The quality of life was significantly increased in six studies that applied: (i) anti-inflammatory and omega 3 diets in patients with rheumatoid arthritis [34,61]; (ii) a plant-based diet and intermittent fasting for musculoskeletal pain [39,62]; and (iii) a vegan/vegetarian diet, a FODMAP diet, and glutamate elimination for fibromyalgia [59,61].

### 4.1. Effects and Benefits of Dietary Strategies on Chronic Musculoskeletal Pain

Studies have shown that the use of nutritional strategies may have an impact on pain and health. In this regard, current research shows that intermittent fasting improves pain perception in patients suffering from chronic pain in the short term. However, other studies have shown these changes to have no significant effect [62]. Caloric restriction has been shown to be a good long term treatment option for people suffering from pain of a rheumatic etiology [60], and a time restricted feeding strategy may serve as a first step towards a caloric restriction diet [20], which has shown both quantitative and qualitative changes in the level of health. These changes include the regulation of the circadian rhythm which are different physiological processes that occur 24 h a day such as blood pressure, the sleep cycle, respiration, heart rate, and cognitive processes [20].

Pain symptoms have been shown to improve by following a diet low in glutamate. High levels of glutamate can produce an excess of oxytocin, which is one of the causes of oxidative stress and neuroinflammation, leading to cell death. The purpose of a low glutamate diet is to reduce exposure to glutamate, while taking in nutrients that protect against an excess of oxytocin and oxidative stress. This type of diet improves the metabolic energy of the neuronal cell, which not only helps to explain improvements in fatigue but also increases the potential for glutamate release in the neurotransmission process [36]. In addition, the use of a Mediterranean diet has been shown to decrease the level of pain [39,41,42], although the contrary is seen when assessing pain frequency and articular stiffness [38,41].

The increased consumption of sugar has become an aggravating factor in health [58] as there is a pro-inflammatory effect that favors an increase in chronic symptoms. Hence, an essential factor in the control and the treatment of arthritis should be to reduce sugar intake [58].

Other nutritional strategies, such as the KD and TRF, have not been found to produce changes in pain perception. However, the majority of these studies were carried out in healthy or overweight and obese adults, and therefore studies using these strategies are needed [20].

### 4.2. Effects and Benefits of Dietary Strategies on Quality of Life in Those Suffering from Chronic Pain

A Mediterranean diet has been shown to positively affect low inflammatory diseases since it is comprised of elements that promote health and regulate certain immunological parameters [58]. Furthermore, it has also been shown to increase the quality of life, sleep wellness, physical, and general well-being. Fasting creates greater tolerance to chemotherapy sessions, as well as positive effects at the metabolic, endocrine, and hematological levels as well as a decrease in DNA damage [29]. Moreover, the addition of exercise to this food strategy reduces insulin resistance and inflammation levels, which positively affects glucose metabolism and increases cognitive functions [27,28], creating beneficial effects on cardiovascular risk factors [28]. Evidence shows that intermittent fasting moderately increases the expression of SIRT3, the main mitochondrial deacetylase, which modulates oxygen production and reduces its damage on cellular components [27].

TRF has been shown to produce quantitative and qualitative changes in the level of health, including the regulation of the circadian rhythm, which represents different physiological processes that occur 24 h a day such as blood pressure, sleep cycle, respiration, heart rate, and cognitive processes. Our knowledge of how meal frequency and timing impact health and the circadian rhythm has yet to be explored, and it needs to be increased [20,63]. In this context, lifestyle factors have also been associated to SNC functioning, brain derived neurotrophic factor, and inflammation, thus highlighting the importance of both assessing and treating patients, including nutritional interventions, within a multimodal treatment [63,64,65].

Finally, the KD seems to improve the quality of life in those suffering from breast cancer, but evidence is scarce and more studies are needed to corroborate these findings [35].

### 4.3. Strengths and Weaknesses of the Study

This review presents a number of strengths. First, the use of different scales to ensure a good methodological quality of the studies collected and, therefore, the results obtained. Second, a wide variety in the studies analyzed, namely RCTs, observational studies, and systematic reviews. Finally, this study exposes current gaps in our understanding of how food strategies affect people suffering from chronic pain, and it proposes new directions to follow in this line. On the other hand, some weak points should be mentioned. The extensive variety of food strategies that can be used in this field of research makes the extrapolation of the results difficult to summarize. Moreover, due to the heterogeneity of the participants the results from this study should be interpreted with caution. Finally, due to the vast number of studies obtained during the search we did not research nor make comparisons between combinations of regimens as we did with the nutritional strategies in isolation.

### 4.4. Clinical Application of the Results and Future Research Lines

Current scientific literature shows that for diseases such as rheumatoid arthritis [34,61], cancer [29], fibromyalgia [59], or chronic musculoskeletal pain [10,40,62], the application of different dietary strategies may be effective. This is due to the fact that it favors the reduction of inflammation levels [9], which lead to a decrease in CNS sensitization. This triggers a decrease in the perception of pain, and therefore an improvement in the quality of life and health of the subjects [10]. Furthermore, the use of nutritional strategies to improve the gut microbiome may justify changes in pain perception and quality of life since poor nutrition has been shown to cause changes in the microbiome that results in inflammation of the systemic and the central nervous system. The chronification and the amplification of pain may be increased by both central and peripheral immune activity, which causes aberrant glial activity and reinforces this mechanism. Based on the results of the presented study, and in order to increase the quality of both research and treatment, several future research lines are proposed: (i) to carry out studies analyzing the efficacy of food strategies on chronic pain and health in combination with other therapies such as exercise and physiotherapy in different conditions, for example chronic pain, cancer, fibromyalgia, cardiovascular, and metabolic diseases; (ii) to analyze the molecular mechanisms that exist behind the benefits produced after the combined application of dietary strategies and other strategies on those patients; and (iii) to analyze changes in oxidative stress biomarkers and inflammatory cell populations, as well as circadian rhythms, after the application of a combined intervention of dietary strategies and other therapies in patients with chronic musculoskeletal pain.

## 5. Conclusions

Studies that show consensus on the effects of an IF diet on pain improvement, in either the short or the long term, are lacking. IF improves mitochondrial metabolism, and with the addition of exercise both insulin resistance and inflammation levels may be decreased, thereby improving glucose metabolism, cognitive functions, and the cardiovascular system.

Caloric restriction is a good long term treatment option for people suffering from pain of a rheumatic etiology. The TRF intervention improves health by affecting the circadian rhythm, and it therefore helps blood pressure, sleep quality, breathing quality, heart rate, and cognitive processes. The KD may improve the quality of life in those suffering from breast cancer.

The quality of the studies collected on dietary strategies in the treatment of chronic pain and health is good. Further studies analyzing the effects of different food strategies on pain, quality of life, the circadian rhythm, and sleep quality, not only in isolation but in combination with other therapies in the short and the long term, are needed.

## Figures and Tables

**Table 1:** Search strategy in the different databases used.

Pubmed	- “Fasting” [MeSH] OR “caloric restriction ” [MeSH] AND “musculoskeletal chronic pain” - “Fasting” [MeSH] OR “caloric restriction ” [MeSH] OR “time restricted”AND “musculoskeletal chronic pain” - “Fasting” [MeSH] AND “musculoskeletal chronic pain” - ((“Fasting”)) AND (Inflammation OR pain OR “musculoskeletal pain” OR “quality of life”) - ((“Ketogenic diet”)) AND (Inflammation OR pain OR “musculoskeletal pain” OR “quality of life”) - ((“Caloric restriction”)) AND (Inflammation OR pain OR “musculoskeletal pain” OR “quality of life”) - ((“Mediterranean diet”)) AND (Inflammation OR pain OR “musculoskeletal pain” OR “quality of life”)
Web of Science	- “Fasting” AND “musculoskeletal chronic pain” - “Fasting” OR “caloric restriction” OR “time-restricted” AND “musculoskeletal chronic pain” - ((“Fasting”)) AND (Inflammation OR pain OR “musculoskeletal pain” OR “quality of life”) - ((“Ketogenic diet”)) AND (Inflammation OR pain OR “musculoskeletal pain” OR “quality of life”) - ((“Caloric restriction”)) AND (Inflammation OR pain OR “musculoskeletal pain” OR “quality of life”)
ProQuest	- “Fasting” OR “caloric restriction” AND “musculoskeletal chronic pain” - (Fasting) AND (“musculoskeletal chronic pain” OR “quality of life”) - (Ketogenic diet) AND (Inflammation OR quality of life OR musculoskeletal chronic pain) - (“Mediterranean diet”) AND (pain) AND (Inflammation OR “musculoskeletal pain” OR “quality of life”)
Scopus	- “Fasting” AND “musculoskeletal chronic pain” - “Fasting” OR “caloric restriction” AND “musculoskeletal chronic pain” - Fasting OR ketogenic diet OR caloric restriction AND inflammation AND musculoskeletal chronic pain AND quality of life - (“Mediterranean diet”) AND (pain) AND (Inflammation OR “musculoskeletal pain” OR “quality of life”)
CINAHL	- “Fasting” OR “caloric restriction” OR “time-restricted” AND “musculoskeletal chronic pain” - Fasting OR ketogenic diet OR caloric restriction AND inflammation AND musculoskeletal chronic pain AND quality of life - (“Mediterranean diet”) AND (pain) AND (Inflammation OR “musculoskeletal pain” OR “quality of life”)
Cambridge Core	- “Fasting” AND “musculoskeletal chronic pain” - “Fasting” OR “caloric restriction” AND “musculoskeletal chronic pain” - (“Mediterranean diet”) AND (pain) AND (Inflammation OR “musculoskeletal pain” OR “quality of life”)
Oxford Academy	- “Fasting” AND “musculoskeletal chronic pain” - “Fasting” OR “caloric restriction” OR “time-restricted” AND “musculoskeletal chronic pain” - Fasting OR ketogenic diet OR caloric restriction AND inflammation AND musculoskeletal chronic pain AND quality of life - (“Mediterranean diet”) AND (pain) AND (Inflammation OR “musculoskeletal pain” OR “quality of life”)

**Table 2:** Study of the methodological quality of the RCTs through the PEDro Scale.

Author, Year	1 *	2	3	4	5	6	7	8	9	10	11	Total
Wegman MP. et al., 2015 [27]	1	1	0	1	1	0	1	1	1	1	1	8/10 Good
Harder-Lauridsen NM et al., 2016 [28]	1	1	0	1	0	0	1	1	1	1	1	8/10 Good
Bauersfeld SP et al., 2018 [29]	1	1	1	1	0	0	0	1	0	1	1	7/10 Good
Liu B. et al., 2019 [30]	0	1	1	0	0	0	1	1	0	1	1	6/10 Good
Cohen CW. et al., 2019 [31]	1	1	0	1	0	0	0	1	0	1	1	5/10 Moderate
Stekovic S. et al., 2019 [32]	1	1	0	1	0	0	1	1	0	1	1	6/10 Good
KhodabakhshiA. et al., 2019 [33]	1	1	0	1	0	0	0	1	1	1	1	6/10 Good
Vadell AKE. et al., 2020 [34]	1	1	0	1	1	0	0	1	1	0	1	6/10 Good
Khodabakhshi A. et al., 2020 [35]	1	1	0	1	0	0	0	1	1	0	1	5/10 Moderate
Holton KF et al., 2020 [36]	1	1	0	1	1	0	1	1	0	1	1	7/10 Good
Che T. et al., 2021 [37]	1	1	1	1	0	1	1	1	1	1	1	9/10 Good

1. The selection criteria were specified. 2. Subjects were randomly assigned to groups. 3. The assignment was hidden. 4. The groups were similar at baseline in relation to the most important prognostic indicators. 5. All subjects were blinded. 6. All therapists who administered the therapy were blinded. 7. All assessors who measured at least one key outcome were blinded. 8. Measures of at least one of the key outcomes were obtained from more than 85% of the subjects initially assigned to the groups. 9. Results were presented for all subjects who received treatment or who were assigned to the control group; or, where this could not be the case, the data for at least one key outcome were analyzed by ‘intention to treat’. 10. Results of statistical comparisons between groups were reported for at least one key outcome. 11. The study provides point and variability measures for at least one key outcome. * Non-summation criterion for the PEDro Scale.

**Table 3:** Internal validity of the selected RCTs.

Author, Year	2	3	5	6	7	8	9	PVI
Wegman MP. et al., 2015 [27]	1	0	1	0	1	1	1	5/7 Moderate
Harder-Lauridsen NM. et al., 2016 [28]	1	0	0	0	1	1	1	4/7 Moderate
Bauersfeld SP et al., 2018 [29]	1	1	0	0	0	1	0	3/7 Limited
Liu B. et al., 2019 [30]	1	1	0	0	1	1	0	4/7 Moderate
Cohen CW. et al., 2019 [31]	1	0	0	0	0	1	0	2/7 Limited
Stekovic S. et al., 2019 [32]	1	0	0	0	1	1	0	3/7 Limited
Khodabakhshi A. et al., 2019 [33]	1	0	0	0	0	1	1	3/7 Limited
Vadell AKE. et al., 2020 [34]	1	0	1	0	0	1	1	4/7 Moderate
Khodabakhshi A. et al., 2020 [35]	1	0	0	0	0	1	1	3/7 Limited
Holton KF et al., 2020 [36]	1	0	1	0	1	1	0	4/7 Moderate
Che T. et al., 2021 [37]	1	1	0	1	1	1	1	6/7 Good

2. Subjects were randomly assigned to groups. 3. The assignment was hidden. 5. All subjects were blinded. 6. All therapists who administered the therapy were blinded. 7. All assessors who measured at least one key outcome were blinded. 8. Measures of at least one of the key outcomes were obtained from more than 85% of the subjects initially assigned to the groups. 9. Results were presented for all subjects who received treatment or who were as‐ signed to the control group; or, where this could not be the case, the data for at least one key outcome were analyzed by ‘intention to treat’. PVI: Internal Validity Score.

**Table 4:** Study of the methodological quality of observational studies through the Quality Assessment Tool for Observational Cohort and Cross-Sectional Studies Scale.

Author, Year	1	2	3	4	5	6	7	8	9	10	11	12	13	14	Total
Veronese N. et al., 2016 [38]	1	1	1	1	1	1	1	0	1	1	1	0	1	1	11/14 Good
Towery P. et al., 2018 [39]	1	1	1	1	1	1	1	0	1	0	1	0	1	1	11/14 Good
Ingegnoli F. et al., 2020 [40]	1	1	1	1	1	1	1	0	0	0	0	0	1	1	9/14 Good
Ortolá R. et al., 2021 [41]	1	1	1	1	1	1	1	0	1	1	1	0	1	0	11/14 Good
Cooper I. et al., 2022 [42]	1	1	1	1	1	1	0	0	1	1	1	0	1	0	10/14 Good

1. The purpose specifically explained. 2. The population studied was clearly explained. 3. The participation rate for eligible individuals was at least 50%. 4. All subjects were recruited from the same or a similar population. 5. The sample size, a description or estimate of the variance and the effect were justified. 6. Exposures of interest were measured before the results were performed. 7. The time period was long enough to reasonably expect to see an association between the exposure and the outcome. 8. The study was examined at different exposure levels with respect to the outcome. 9. Exposure measures were clearly defined, valid, reliable, and consistently implemented in the participants. 10. Exposures were evaluated more than once over time. 11. Outcome measures were clearly defined, valid, reliable, and consistently implemented in the participants. 12. Outcome assessors were blinded to the exposure status of the participants. 13. Loss to follow-up after the start of the study was less than 20%. 14. Key confounders were measured and statistically adjusted for their impact on the exposure–outcome relationship.

**Table 5:** Development of the general characteristics of the selected studies.

Author, Year	Type of Study	Experimental Group Control Group Samples	Interventions	Variables	Results
Wegman MP. et al., 2015 [27]	Randomized clinical trial	EG: 12 people Age range men: 22.1–30.05 Average age of men: 22.82 Age range women: 20.05–30.08 Average age women: 24.05 Average BMI for men: 25.32 Average BMI women: 22.77 CG: 12 people Age range of men: 22.1–30.05 Average age of men: 22.82 Age range of women: 20.05–30.08 Average age of women: 24.05 Average BMI for men: 25.32 Average BMI for women: 22.77	EG: progressed to intermittent fasting for 3 weeks CG: for 3 weeks, progressed to intermittent fasting and intake of antioxidant supplements	Satisfaction with diet Genetic expression Oxidative stress level Plasma insulin level	Patients had good adherence and tolerance to fasting. There were no major weight changes. Decrease in the level of oxidative stress and no significant change in gene expression, but *SIRT3* trended towards significance (*p* = 0.0772). Metabolic changes emerged. Intermittent Fasting decreased plasma insulin (*p* = 0.0023).
Veronese N. et al., 2016 [38]	Cohorts study	N = 4470 (2605 women & 1865 men)	All participants had their adherence to the Mediterranean diet (MD) and their quality of life measured by SF-12. Disability, pain, and stiffness were measured by the Western Ontario and McMaster Universities Arthritis Index (WOMAC) and depressive symptoms were assessed with the Center for Epidemiologic Studies Depression Scale (CES-D).	Adherence to Mediterranean diet Disease impact Depressive symptoms Quality of life	A higher MD was significantly associated with a higher SF-12 scale value (b: 0.10; 95% CI: 0.05, 0.15; *p =* 0.0001), lower WOMAC scores, except for stiffness, and lower CES-D scores (b: 20.05; 95% CI: 20.09, 20.01; *p* = 0.01).
Harder-Lauridsen NM. et al., 2016 [28]	Randomized clinical trial	EG: 10 healthy people CG: 10 healthy people	EG: during alternate days they were served 175% (4 meals) or 25% (1 meal). The fasting days were 16–19 h CG: they received 100% (3 meals)	VO2 max Glycemic control MRI Cholesterol Glucose levels	There were no significant differences between groups in the energy obtained from food, in the glycemic control, in the MRI (0.047). Systolic pressure increased in the experimental group (*p* = 0.04), as well as their plasma glucose level decreased in the mornings after fasting (*p* = 0.34)
Bauersfeld et al., 2018 [29]	Randomized clinical trial	EG: 18 women diagnosed withgynecological cancer Age range: 28–63 years old Mean age of diagnosis: 49.8 ± 9.1 years CG: 16 women diagnosed with gynecological cancer Age range: 44–69 years old. Mean age of diagnosis: 53.6 ± 7.3 years	EG: fasted while receiving the first three chemotherapy sessions. During the last three they received normocaloric nutrition. CG: they followed normocaloric nutrition during the first three chemotherapy sessions. During the last three they fasted.	Pain intensity (FACT-G). Fatigue intensity (FACIT-F) Fatigue intensity (FACIT-TOI)	There were no significant differences between the two groups in the FACT-G results, only in social/family well-being (*p* = 0.042). In group A there were significant changes during fasting with respect to chemotherapy received with normocaloric nutrition (mean 12.8). Group B did not have any significant change between both cycles of chemotherapy. They found changes of QQL (mean difference 4.6).
Towery P. et al., 2018 [39]	Observational study	20 people with chronic musculoskeletal pain.	For 8weeks they went on a plant-based diet.	Limitation on ACV Pain (SF-36) Quality of life	The level of pain decreased by 3.14 points out of 10 on the scale (*p* = 0.0001). The SF-36 scores improved from 57.5 to 82.5 (*p* = 0.0001). Weight decreased on average by 1.207 kg (*p* = 0.895).
Liu B. et al., 2019 [30]	Randomized clinical trial	EG 1: 25 women diagnosed with obesity. Age range: 35–70 years old. EG 2: 25 women diagnosed with obesity. Age Range: 35–70 Years EG 3: 26 women diagnosed with obesity. Age Range: 35–70 Years CG: 12 women diagnosed withobesity. Age range: 35–70 years old.	For 8 weeks: CG: maintained the same diet. EG 1: carried out 70% caloric restriction. EG 2: performed 70% intermittent fasting. EG 3: performed 100% intermittent fasting.	Metabolic glucose Tissue inflammation. Fat tissue and muscle.	There was no largely significant difference in metabolism between groups (*p* = 0.05). The size of adipocytes decreased in all groups (*p* = 0.01). Insulin sensitivity appeared to be altered in group 3 (*p* = 0.06). The levels of inflammation in the tissues and muscles increased after the meal sessions (*p* = 0.02).
Cohen CW. et al., 2019 [31]	Randomized clinical trial	EG: 23 women with ovarian or endometrial cancer. CG: 20 women with ovarian or endometrial cancer.	EG: for 12 weeks they followed a Ketogenic diet: 70% fat, 25% protein and 5% carbohydrate. CG: for 12 weeks they followed the dietary requirements established by the ACS and the Academy of Nutrition and Dietetics for cancer patients and survivors.	Cholesterol Β-hydroxybutyrate Colorimetric assay	There were no significant differences in total cholesterol, HDL-C, LDL-C, TC:HDL-C ratio or TG:HDL-C ratio between groups. The ACS group decreased fasting triglycerides (*p* < 0.05) and TG:HDL-C levels (*p* < 0.05). The KD group had higher Β-hydroxybutyrate concentrations (*p* < 0.001). Significant difference between groups in total energy expenditure (*p* < 0.01). Between-group differences in total carbohydrates, total protein, saturated fat, cholesterol, dietary fiber, and total sugar were observed (*p* < 0.0001).
Stekovic S. et al., 2019 [32]	Randomized clinical trial	EG: 30 people CG: 30 people	EG: for 4 weeks alternate days of intermittent fasting and days of standardized nutrition. CG: performs standardized nutrition for 4 weeks.	Levels of inflammation Blood in plasma Cholesterol Oxidative stress	Experimental group reduced body weight, as well as the risk of cardiovascular disease (*p* < 0.0001). There were no differences in energy activity levels between both groups (*p* = 0.258). The levels of inflammation were significantly lower over a long period of time (*p* = 0.04).
Khodabakshi A. et al., 2019 [33]	Randomized clinical trial	EG: N = 40; Mean age = 44.8 ± 8.4 CG: N = 40; Mean age = 45.2 ± 15.0 Both groups have patients with a malignant biopsy and undergoing chemotherapy for at least 3 months.	EG: they follow a Ketogenic diet (6% carbohydrate, 19% protein, 20% medium chain triglycerides, and 55% fat) for 90 days. CG: they follow a regular diet (55% carbohydrate, 15% protein and 30% fat) for 90 days.	Body weight BMI Body fat Fasting blood sugar Ketone bodies TG ALT AST BUN Cr Level HDL-LDL Cholesterol	The levels of fasting blood sugar increased in the EG (*p* <0.001). The EG reduced body-weight (*p* < 0.001). There were significant differences between groups in weight, BMI, and body fat (*p* < 0.001, *p* <0.001, *p* = 0.03). TG, AST, Cr Level, and BUN were different between groups (*p* = 0.04, *p* = 0.04, *p* = 0.01, *p* = 0.04).
Vadell AKE. et al., 2020 [34]	Randomized clinical trial	EG: 26 people with a diagnosis of Rheumatoid Arthritis for ≥2 years. Age range: 18–75 years old. Residence: Sweden. CG: 24 people with a diagnosis of Rheumatoid Arthritis for ≥2 years. Age range: 18–75 years old. Residence: Sweden.	There were two periods of 10 weeks (the groups exchanged intervention). EG: performed an anti-inflammatory nutrition. CG: performs standardized nutrition.	Levels of inflammation Quality of life	There was no difference between energy intake and weight levels (*p* = 0.082) during the separate diets (median: −0.4 kg; IQR: −1.4, 0.6 kg during the intervention period; *p* = 0.082 and median: 0.3 kg; IQR: −0.7, 1.6 kg during the control period; *p* = 0.122). The experimental group presented a reduction in the concentration of anti-inflammatory indicators.
Ingegnoli F. et al., 2020 [40]	Observational, cross-sectional, single-center study	N = 205 (165 women & 40 men)	All participants had their adherence to a Mediterranean diet and their score of various scales related to their quality of life and the impact of their disease recorded.	Adherence to Mediterranean diet Impact of their disease Quality of life	An association between the Mediterranean Diet score and the Health Assessment Questionnaire (*p*-value = 0.033) and patient global assessment and general health (*p*-value = 0.023 in both) was observed. Rheumatoid Arthritis Impact of Disease total score had a statistically significant negative relationship with the Mediterranean Diet score (*p*-value = 0.016). A statistically significant negative association was found for pain (*p*-value = 0.025), functional disability (*p*-value < 0.001), sleep (*p*-value = 0.041), physical well-being (*p*-value = 0.027), and coping (*p*-value = 0.008).
Khodabakhshi A. et al., 2020 [35]	Randomized clinical trial	EG: N = 40 CG: N = 40 Both groups comprised of patients between 18 and 70 years of age who had breast cancer with locally advanced or metastatic disease, and who were receiving chemotherapy for at least 12 weeks.	EG: they followed a Ketogenic diet (6% carbohydrates, 19% protein, 20% medium-chain triglyceride and 55% fat) for 12 weeks. CG: they followed a standard diet (55% carbohydrates, 15% protein and 30% fat) for 12 weeks.	Biomarkers (Na^+^, K^+^, Ca^++^, P^+^, Lactate, Mg^++^, LDH, Albumin, Ammonia, ALP) Quality of life (EORTCQLQ-C30, IORTCQLQ-BR23)	The EG had a better global Quality of Life (*p* = 0.02). Diarrhea increased in CG (*p* = 0.02). There was an increase in physical performance measures in the EG (*p* = 0.04). The CG had a significant decrease in role functioning and social functioning (*p* = 0.02, *p* = 0.02).
Holton et al., 2020 [36]	Randomized clinical trial	EG: 20 people with Gulf War Syndrome. Average age: 54.35 ± 6.02 years. BMI: 32.10 ± 5.34 kg/m^2^ CG: 20 people with Gulf War Syndrome. Average age: 54.35 ± 6.02 years. BMI: 32.10 ± 5.34 kg/m^2^	EG: followed a low glutamate diet for a month. CG: they did not make any changes in their type of diet.	Intensity and frequency of pain.	There were severe changes in the intensity and frequency of symptoms. The mean evaluation of symptoms in the experimental group was 11.7 ± 5.3, and in the control group it was 18.1 ± 5.7.
Ortolá R. et al., 2021 [41]	Cohorts study	N = 1726 (1091 women & 635 men)	Adherence to the Mediterranean diet was measured with the Mediterranean Diet Adherence Score (MEDAS) at baseline. Frequency, severity, and location of pain from baseline and follow-up were used to compute a pain scale.	Adherence to the Mediterranean diet Pain characteristics	Participants with the lowest adherence to the Mediterranean diet showed a lower frequency of pain improvement (relative risk ratio [95% confidence interval]: 1.43 [1.03, 1.99]). This was also evidenced by an improvement in pain severity (1.43 [1.01, 2.04]) and a reduction in pain location (1.54 [1.08, 2.20]). A tendency to pain frequency improvement (1.34 [0.92, 1.93]) was also observed.
Che T. et al., 2021 [37]	Randomized clinical trial	EG: N = 60; Mean age = 48.21 ± 9.32; Female = 29; Male = 31 CG: N = 60; Mean age = 48,78 ± 9.56; Female = 26; Male = 34 Both groups were comprised of people with type 2 diabetes.	EG: they followed Time-restricted feeding (ate freely from 8:00 to 18:00 and fasted from 18:00 to 8:00) for 14 weeks. CG: they followed a regular diet for 14 weeks.	Biomarkers: (Hemoglobin, Fasting plasma glucose (FPG), Β-cell function (HOMA-β), Insulin resistance (HOMA-IR), Cholesterol). Body weight and BMI Adherence Physical activity (step count)	The difference in average eating was not notable (*p* = 0.62). The eating window reduced significantly (*p ≤* 0.001). The EG had a significant reduction in Hemoglobin (*p* < 0.001), FPG (*p* < 0.001), body weight (*p* < 0.001), BMI (*p* < 0.001), HOMA-IR (*p* = 0.02) and HOMA-β (*p* = 0.005). The total step count was similar in both at 12 weeks (*p* = 0.62)
Cooper I. et al., 2022 [42]	Single-arm feasibility trial	N = 28 (23 women & 5 men) Intervention: 22 Losses: 6	All participants followed a 9-week anti-inflammatory diet, which consisted of minimal processed foods and higher amounts of “good” fats and whole foods. They were encouraged to consume a normocaloric diet to satiety.	Knee symptoms Health-related quality of life Body mass	The Knee Injury and Osteoarthritis Outcome Score reported an improvement in the results supported by the minimal detectable change (MDC) in 8–10. EuroQoL-5D reported a non-significant variation in the quality of life of the participants. Participants lost an average of 3 kg, but it was not a significant difference.

## Data Availability

Data can be requested from the corresponding author.

## Abbreviations

ADL	Activities of Daily Living
AIMS2-SF	Arthritis Impact Measurement Scales 2
ALT	Alanine Transminase
AST	Asparate Aminotransferase
BMI	Body mass index
BUN	Blood Urea Nitrogen
CG	Control Group
CINAHL	Cumulative Index to Nursing & Allied Health Literature
CNS	Central Nervous System
DHA	Docosahexaenoic Acid
DNA	Deoxyribonucleic Acid
EG	Experimental Group
EPA	Eicosapentaenoic Acid
FACT-G	Functional Assessment of Cancer Therapy—General
FSQ	Fibromyalgia Survey Questionnaire
HDL	High Density Lipoprotein
HRQL	Generic health-related quality of life
IVS	Internal Validity Score
LDL	Low Density Lipoprotein
MeSH	Medical Subject Headings
NPRS	Numeric Pain Rating Scale
PRISMA	Preferred Reporting Items for Systematic Reviews and Meta-Analyses
RCTs	Randomized Clinical Trials
RMI	Magnetic Resonance Imaging
SF-36	Health Questionnaire
SIRT3	Sirtuin 3
SSS	Symptom Severity Scale
TC	Total Cholesterol
TG	Triglyceride
VAS	Visual analogue Scale
VO2 Max	Maximum Oxygen Volume
WPI	Widespread Pain Index
WOMAC	Western Ontario and McMaster Universities Osteoarthritis Index
